# Association of coarctation of aorta with Turner syndrome: a case report

**DOI:** 10.3389/fped.2025.1607621

**Published:** 2025-08-14

**Authors:** Musawer Khan, Sana Imtiaz, Muhammad Shoaib, Malaika Tariq, Sadia Zahra, Awais Sardar, Asif Ullah Khan, Kamil Ahmad Kamil

**Affiliations:** ^1^ Department of Medicine, Combined Military Hospital Quetta, Quetta, Pakistan; ^2^ SMBZAN Institute of Cardiology, Quetta, Pakistan; ^3^ Department of Pediatrics, CMH Quetta, Quetta, Pakistan; ^4^ Quetta Institute of Medical Sciences, Quetta, Pakistan; ^5^ Department of Pediatrics, Combined Military Hospital Quetta, Quetta, Pakistan; ^6^ Internal Medicine Department, Mirwas Regional Hospital, Kandahar, Afghanistan

**Keywords:** Turner syndrome, coarctation of aorta, karyotyping, echocardiography, congenital heart disease, balloon angioplasty, case report

## Abstract

**Background:**

Monosomy 45,X is commonly associated with congenital heart defects, particularly coarctation of the aorta (CoA). In this case, the patient developed respiratory distress due to hemodynamic instability from a large bidirectional patent ductus arteriosus (PDA) shunt and systemic hypoperfusion secondary to CoA, which complicated diagnosis and management.

**Case presentation:**

We report a 34-week premature female neonate weighing 1.94 kg, delivered via lower segment cesarean section (LSCS) due to oligohydramnios and intrauterine growth restriction. She exhibited characteristic features of Turner syndrome, including a webbed neck, low-set ears, widely spaced nipples, and lymphedema of the hands and feet. Karyotyping confirmed a 45,X monosomy. Echocardiography revealed a bicuspid aortic valve, juxtaductal coarctation of the aorta, a moderate-sized PDA with a bidirectional shunt, and suspected pulmonary hypertension. A contrast-enhanced CT aortogram confirmed the coarctation. The patient was managed with mechanical ventilation, continuous positive airway pressure (CPAP), surfactant therapy, and phototherapy. Rescue transcatheter balloon angioplasty was performed for the coarctation, followed by PDA ligation and surgical coarctation repair at a tertiary center, resulting in marked clinical improvement.

## Introduction

Turner syndrome (TS) is a genetic condition resulting from complete or partial loss of one sex chromosome in females, with an incidence of 1 in 2,500 live-born female infants. It is associated with various multisystem anomalies, among which congenital heart anomalies are most common, particularly coarctation of aorta (CoA), bicuspid aortic valve (BAV), and aortic stenosis (AS) (1).

Studies have shown that in patients with Turner syndrome, CoA is found in one-third of patients while in 25% of cases, BAV is prevalent (2). Turner syndrome presents with multiple sets of clinical features including primary hypogonadism, skeletal anomalies, congenital heart disease, gastrointestinal and hepatic dysfunctions, visual and auditory problems, lymphatic and cutaneous complications, renal malformations, and mental deficits (3). Despite being well-documented, the occurrence of CoA, PDA, and pulmonary hypertension in neonates altogether results in serious hemodynamic instability, presenting a unique range of diagnostic and therapeutic challenges. Turner syndrome can be suspected on routine ultrasound; however, diagnosis can be confirmed by prenatal karyotyping through amniocentesis and chorionic villi sampling (4).

### Patient information

A 34-week preterm female neonate was delivered via lower segment cesarean section (LCS) at CMH Quetta on 11 November 2024 due to oligohydramnios and intrauterine growth restriction (IUGR), with a maternal history of gestational diabetes mellitus (GDM) and thalassemia trait. The baby had a low birth weight of 1.94 kg and fronto-occipital circumference (FOC) of 30 cm, with good APGAR scores.

### Upon admission

#### Vitals signs

Pulse 160 bpm, respiratory rate 65/min, and blood pressure 68/45 mmHg. She was admitted to the NICU immediately after birth due to respiratory distress.

#### On physical examination

The neonate was hypotonic with reduced spontaneous movement. She had dysmorphic features including a webbed neck, low-set ears, widely spaced nipples, and lymphedema of hands and feet.

Cardiovascular examination revealed a prominent precordial impulse, bounding peripheral pulses, reveals large pulmonary component of second heart sound (loud P2), and a continuous machinery murmur best heard at the left upper sternal border.

Respiratory examination showed tachypnea, nasal flaring, and intercostal retractions. Abdominal exam was soft with no organomegaly, and neurologically, the baby was lethargic but responsive to stimuli.

### Timeline

The patient was admitted on 11 November 2024 and immediately transferred to the NICU due to respiratory distress. The first echocardiogram was performed on 25 November 2024, followed by a second on 9 December 2024. A contrast-enhanced CT aortogram was conducted on 10 December 2024, confirming juxtaductal coarctation. Rescue transcatheter balloon angioplasty was performed on 12 December 2024, with a follow-up echocardiogram on 13 December 2024 showing improvement. The patient was transferred to a tertiary cardiac institute on 6 January 2025, where she underwent successful PDA ligation and coarctation repair on 29 January 2025.

## Clinical finding

Initial laboratory investigations revealed a hemoglobin level of 18.3 g/dL, total leukocyte count of 9.9 × 10^9^/L, platelet count of 265 × 10^9^/L, and C-reactive protein of 1 mg/L. Serum sodium was 143 mmol/L, potassium 5.2 mmol/L, calcium 1.82 mmol/L, urea 6.0 mmol/L, creatinine 87 µmol/L, albumin 36 g/L, ALT 10 U/L, and serum total bilirubin 110 µmol/L. Magnesium was 0.8 mmol/L, phosphate 2.66 mmol/L, lactate 4.7 mmol/L, and TSH 2.78 mIU/L. The Coombs test was negative. These findings supported stable general metabolic status, with no evidence of significant infection or metabolic derangement.

### Diagnostic assessment

#### Imaging

**Figure 1:**
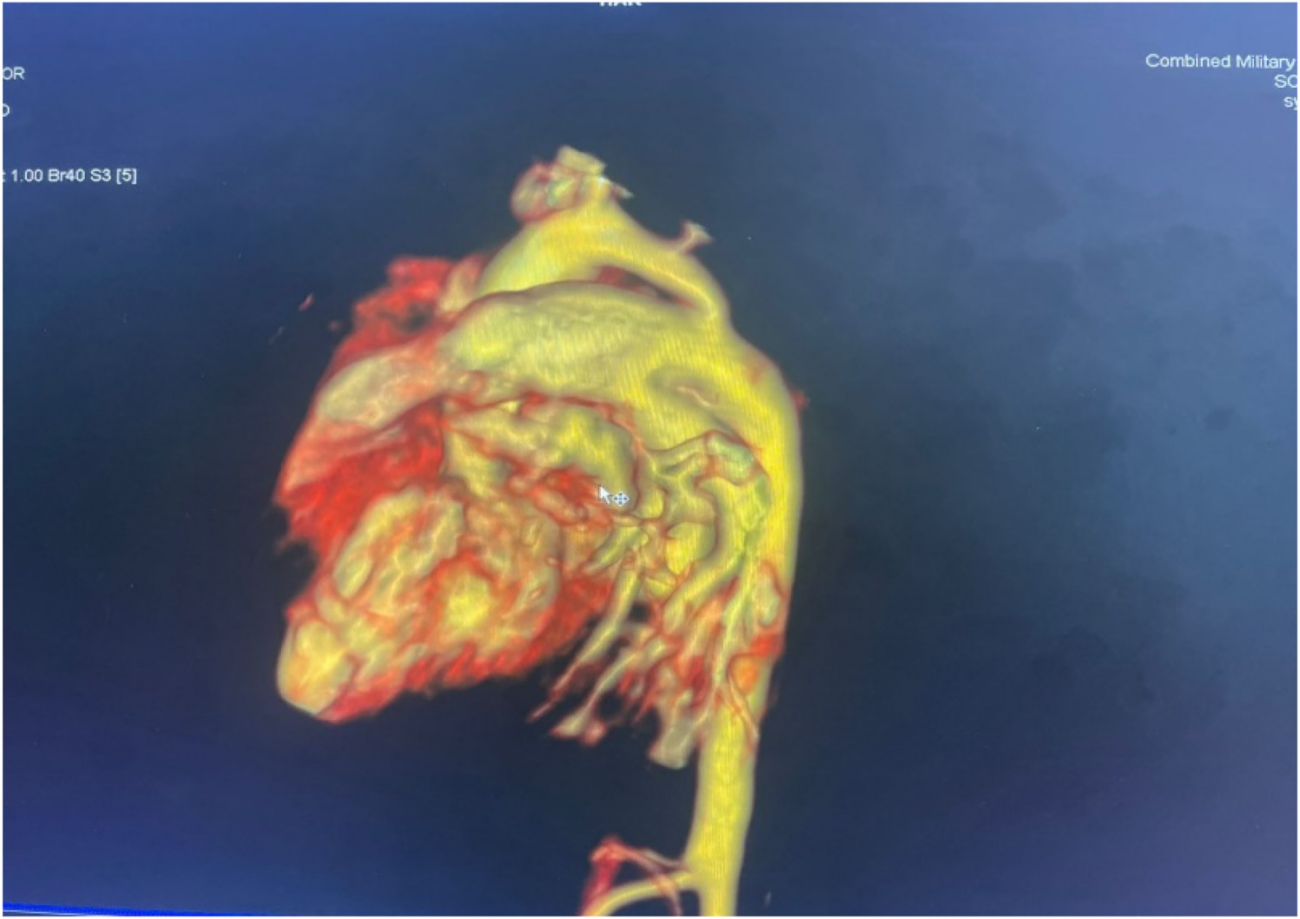
Volume-rendered 3D reconstruction of a CT aortogram showing severe discrete coarctation with a large PDA continuing into the descended aorta.

**Figure 2:**
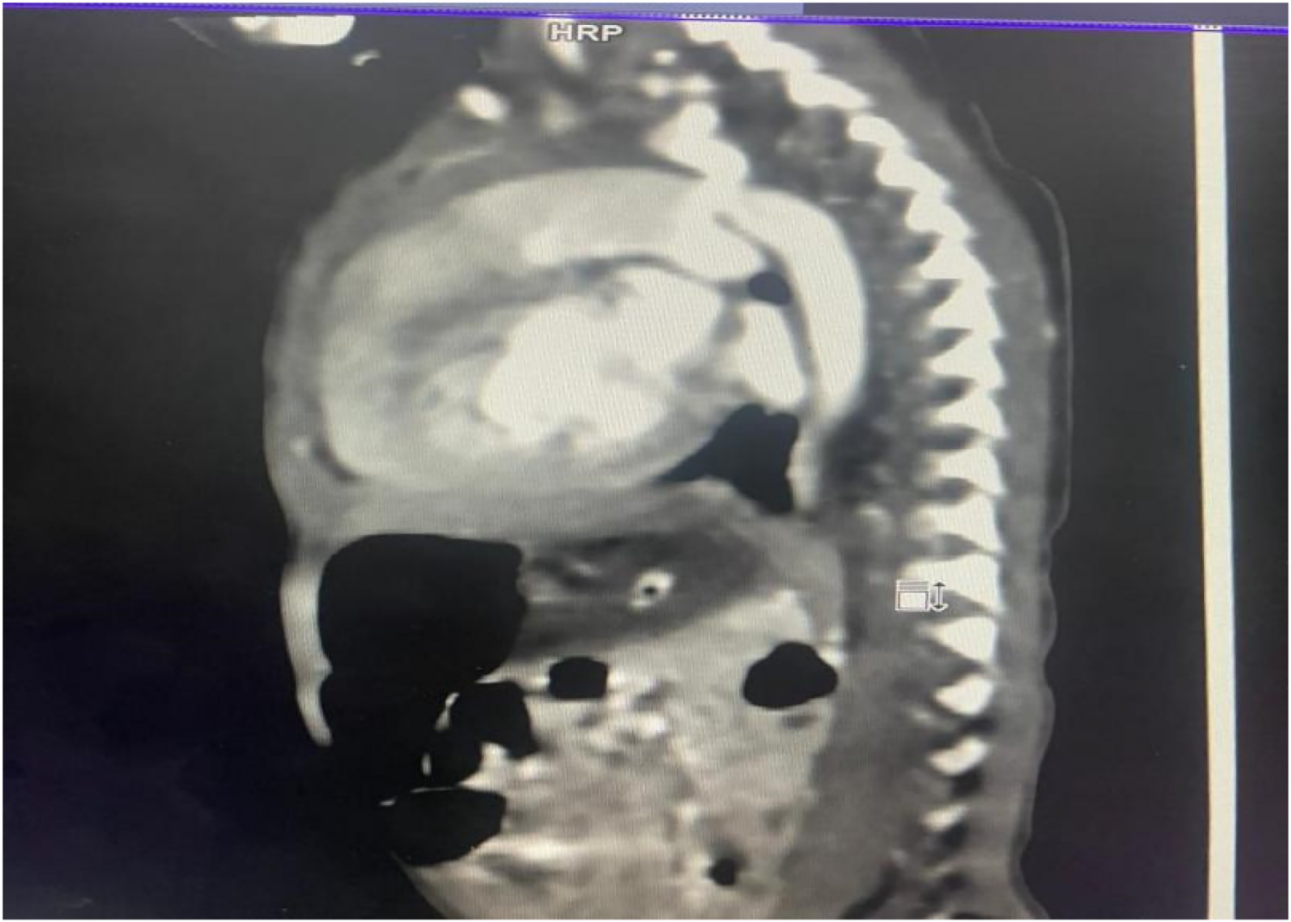
CT aortogram sagittal view after ballooning.

For imaging, see Figures 1 and 2 and Table 1.

**Table 1:** Summary of diagnostic imaging findings.

Chest x-ray	Shows cardiomegaly, increased pulmonary vascular marking
2D echocardiography 1st	The Bicuspid aortic valve, mild coarctation of the aorta, moderate size patent ductus arteriosus (PDA) bidirectional shunt
2D echocardiography 2nd	Shows Worsening of coarctation, severe PAH, large PDA bidirectional shunt, patent foramen ovale, and bicuspid aortic valve
CECT aortogram	Confirmed juxtaductal coarctation of aorta, large PDA, bicuspid pulmonary valve, borderline cardiomegaly
Postprocedure (coronary angioplasty) 2D echo	Revealed a significant decrease in coarctation gradient with left to right shunts direction across PDA and left ventricle hypertrophied with good function
Postprocedure (PDA ligation and coarctation of aorta repair) 2D echo	Shows no gradient across the descending aorta and good left ventricular function
Karyotyping	G band chromosome analysis shows an abnormal karyotype. All the metaphases reveal 45X with clinical diagnosis of Turner syndrome

2D echo, two-dimensional echocardiography; CECT, contrast-enhanced computed tomography; PDA, patent ductus arteriosus.

### Diagnostic assessments

The diagnostic workup included a chest radiograph showing cardiomegaly with increased pulmonary vascular markings. Echocardiography confirmed a bicuspid aortic valve, juxtaductal coarctation of the aorta, and a moderate-sized PDA with a bidirectional shunt. A follow-up echocardiogram demonstrated worsening coarctation and suspected pulmonary hypertension. A CT angiogram provided detailed anatomical confirmation of the coarctation and PDA. Although pulmonary hypertension was suspected based on echocardiographic findings and clinical signs such as respiratory distress and a loud P2, we acknowledge that in neonates with critical left-sided obstructive lesions like CoA, reduced systemic pressures can mimic elevated pulmonary pressures. Direct catheter-based pressure measurements were not feasible in our setting, and echocardiographic estimates were limited by technical challenges. The hemodynamic compromise was thus interpreted as multifactorial, primarily due to ductal steal physiology and systemic hypoperfusion from severe coarctation rather than isolated pulmonary arterial hypertension.

### Differential diagnosis

The diagnostic workup included Noonan Syndrome, 46XX Gonadal Dysgenesis, Takayasu Arteritis, Hypoplastic Left Heart Syndrome.

### Therapeutic intervention

Respiratory distress was initially managed with CPAP ventilation. An intravenous line was secured, first-line antibiotics were initiated, and orogastric feeding was started. Management followed the transient tachypnea of the newborn protocol, with surfactant administration as needed. Cardiac issues were initially treated with low-dose diuretics and fluid restriction. Jaundice developed on day 1 of life and was successfully managed with standard phototherapy.

Despite 15 days of non-invasive ventilation, the infant’s respiratory distress worsened, necessitating mechanical ventilation. As congenital cardiac facilities were not available at our center, rescue transcatheter coarctation ballooning was performed on day 32 of life by a pediatric cardiologist via the femoral artery approach. A 5 × 15 mm coronary angioplasty balloon was used for the procedure. Postprocedure echocardiography showed a significant reduction in the coarctation gradient, with a left-to-right shunt across the PDA and a hypertrophied but well-functioning left ventricle. After the procedure, the infant showed marked clinical improvement, with reduced oxygen requirements and respiratory distress.

On day 57 of life, the patient was successfully weaned off oxygen support and referred to the tertiary care cardiac institute for coarctation repair. PDA ligation surgery was successfully performed on day 80 of life.

### Follow-up and clinical outcome

On follow-up, the patient is doing well. Echocardiography showed no gradient across the descending aorta and preserved left ventricular function. The parents were advised to perform hemoglobin electrophoresis at 6 months of age and to ensure regular endocrinology follow-ups for thyroid function and growth monitoring.

## Discussion

Congenital heart diseases occur in 25%–50% of individuals with Turner syndrome. Among these, BAV is nearly 60 times more common than in the general female population (4), while CoA affects approximately 7%–18% of affected individuals (5).

In this case, a preterm neonate presented with features suggestive of Turner syndrome and was found to have BAV, CoA, and a moderate PDA. The initial clinical suspicion of pulmonary arterial hypertension (PAH) was based on echocardiographic findings of a bidirectional PDA and clinical signs such as a loud P2. However, in neonates with critical left-sided obstruction, such as CoA, a bidirectional or right-to-left shunt across a PDA may result from low systemic postductal pressure rather than elevated pulmonary arterial pressure (5). In our resource-limited setting, invasive hemodynamic assessment was not possible, and echocardiographic estimates (e.g., tricuspid regurgitation gradient and pulmonary acceleration time) were not reliably obtainable due to technical limitations. Therefore, PAH in this case is best interpreted as suspected rather than confirmed.

One of the key management decisions in this case was the use of transcatheter balloon angioplasty as a bridge to definitive surgical repair (6). Although surgery is typically preferred for CoA in neonates, balloon angioplasty has been employed in selected cases where immediate surgical intervention is not feasible or when clinical instability requires urgent decompression of the aortic gradient. In our context, where pediatric cardiac surgery was unavailable and timely referral was challenging, this approach provided temporary stabilization and allowed successful weaning from mechanical ventilation.

This case highlights the importance of early diagnosis and a multidisciplinary approach, especially in low-resource settings. Despite the complexity of the case and limitations in diagnostic precision, clinical stabilization was achieved through prompt supportive care and staged intervention. Long-term follow-up will be essential to monitor for complications such as restenosis, aneurysm formation, and hormonal issues related to TS, including the initiation of estrogen replacement therapy (ERT) and growth monitoring (2, 7).

This case also highlights the successful use of balloon angioplasty as a bridge to definitive surgical correction. However, a key limitation was the lack of invasive hemodynamic assessment to confirm pulmonary arterial hypertension, relying instead on echocardiographic estimates, which may be confounded by severe aortic obstruction and PDA shunting.

## Conclusion

This case underscores the importance of integrated care, proactive management, and comprehensive follow-up in TS patients with multiple congenital heart diseases, essential in improving survival and quality of life.

## Data Availability

The original contributions presented in the study are included in the article/Supplementary Material, further inquiries can be directed to the corresponding author.
