# Supplementary figures and images for: Association of coarctation of aorta with Turner syndrome: a case report
Source: Front Pediatr. 2025 Aug 14;13:1607621. doi: 10.3389/fped.2025.1607621 (PMC12406220; doi:10.3389/fped.2025.1607621)

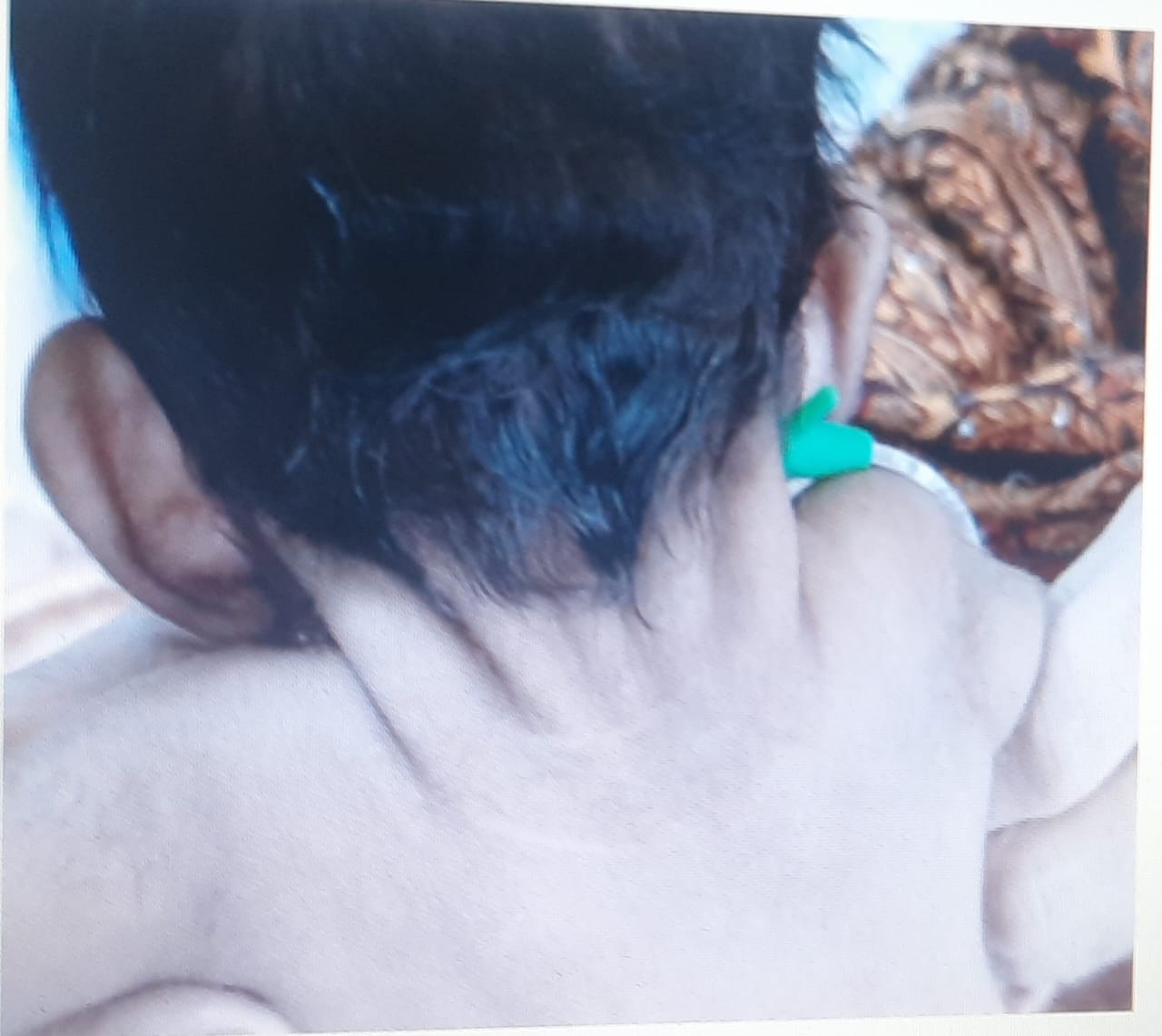

Supplement: Supplementary file 2 [file Image1.jpeg]
